# Mapping of quantitative trait loci for root hair length in wheat identifies loci that co-locate with loci for yield components

**DOI:** 10.1093/jxb/erw228

**Published:** 2016-06-16

**Authors:** R. Horn, L. U. Wingen, J. W. Snape, L. Dolan

**Affiliations:** ^1^Department of Crop Genetics, The John Innes Centre, Norwich NR4 7UH, UK; ^2^Department of Plant Sciences, University of Oxford, South Parks Road, Oxford OX1 3RB, UK

**Keywords:** Mapping, QTL, root hairs, wheat, yield.

## Abstract

Root hairs are fast growing, ephemeral tubular extensions of the root epidermis. They arise in the unsuberized maturation zone of the root, effectively increasing the root surface area in the region over which nutrient and water uptake occur. Variation in root hair length (RHL) between varieties has been shown to be genetically determined, and could, therefore, have consequences for nutrient capture and yield potential in crops. We describe the development of a medium-to-high throughput screening method for assessing RHL in wheat at the seedling stage. This method was used to screen a number of wheat mapping population parental lines for variation in RHL. Parents of two populations derived from inter-varietal crosses differed for RHL: Spark *vs* Rialto and Charger *vs* Badger. We identified quantitative trait loci (QTLs) for RHL in the populations derived from these crosses. In Spark × Rialto, QTLs on chromosomes 1A, 2A and 6A were associated with variation in RHL, whilst in Charger × Badger, a QTL for RHL was identified on 2BL. The QTLs on 2A and 6A co-localized with previously described QTLs for yield components. Longer root hairs may confer an advantage by exploiting limiting mineral and water resources. This first QTL analysis of root hair length in wheat identifies loci that could usefully be further investigated for their role in tolerance to limiting conditions.

## Highlight

Quantitative trait loci for wheat root hair length co-locate with those previously described for harvest index and thousand grain weight.

## Introduction

Root hairs greatly increase the effective surface area of the root in the root zone where the majority of the water and nutrients occur (Doussan *et al.*, 1998; Sanderson *et al.*, 1988), yet their contribution to yield in wheat has been largely overlooked. Grain yield under drought conditions is determined by the amount of water the plant can extract from the soil and its subsequent conversion to biomass and partitioning to grain (Foulkes *et al.*, 2002). Yield is limited by nutrient deficiency, both directly and as a result of increased vulnerability to biotic factors. Wheat is increasingly being produced under suboptimal conditions, where irrigation, fertilization, or both are economically and practically non-viable. Current and projected increases in demand for cereal production necessitate the development of wheat varieties with improved yield on drought stricken and impoverished soils. This is due to a number of factors including environmental and man-made depletion of agronomic soils, mismanagement of existing land, climate change, and demand-led increases in use of low-grade agronomic land (FAO, 2009). Even prime agricultural land can suffer from low nutrient status due to the immobilization of nutrients with low diffusion rates such as phosphate and iron, as well as loss of fertilizer from surface run-off, leaching, remobilization by soil biota, and gaseous plant emission (Raun, 1999).

Root hairs are fast growing, short-lived, tubular, unicellular outgrowths of the root epidermis that serve to interact with the soil and mycorrhizae as well as to anchor the plant (Stolzy and Barley, 1968). By greatly increasing the effective radial surface area of the root, root hairs increase the volume of the soil from which immobile nutrients can be absorbed (Gahoonia and Nielsen, 1997; Bates and Lynch, 2000*a*; Parker *et al.*, 2000; Foreman and Dolan, 2001). The structure of the hairs means that they can forage between the soil particles, creating a diffusion gradient between the root and hair tip, intercepting moisture and nutrients (Segal *et al.*, 2008). Root hairs are produced at the actively growing extremities of the root system in response to environmental cues, and thus they require less investment in biomass than other strategies for increasing soil contact such as producing longer or thicker roots. Additionally, there is less mechanical resistance to the growth of hairs through the soil than to the growth of larger structures (Bates and Lynch, 2000*b*). Root hairs have also been shown to confer an advantage in terms of soil penetration into high-strength soil layers, which is particularly relevant to modern low or no-till practices (Haling *et al.*, 2013).

The final hair length is determined by plant species and genotype (Dittmer, 1947, 1949; Bole, 1973; Gahoonia and Nielsen, 1997) and is influenced by a number of environmental factors, including the availability of nutrients and water. Phosphate (Föhse *et al.*, 1991; Gahoonia *et al.*, 1997), iron (Schmidt and Schikora, 2001), manganese, and zinc (Ma *et al.*, 2001) have all been shown to affect both length and density of root hairs. Of these, variation for phosphate uptake has been best described. In barley, cultivar-specific variation in root hair length has been shown to directly relate to the size of zones of P depletion in the soil (Gahoonia and Nielsen, 1998). In another study Gahoonia and Nielsen (2004) showed that cultivars with longer root hairs were able to maintain their yields under low soil P, whilst shorter haired cultivars suffered a yield penalty. Haling *et al.* (2013) showed that root hairs confer an advantage for P uptake in unfertilized high-strength soils, and low-strength fertilized soils, although not high-strength fertilized soils.

Research in a variety of species including Arabidopsis (Bates and Lynch, 1996; Ma *et al.*, 2001; Schmidt and Schikora, 2001), barley (Gahoonia *et al.*,1997; Gahoonia and Nielsen, 1997, 2004), soyabean (Wang *et al.*, 2004), wheat (Ewens and Leigh, 1984), and common bean (Yan *et al.*, 2004), as well as investigations comparing multiple species (Dittmer, 1947, 1949), has demonstrated that root hair length (RHL) is both genetically determined and environmentally responsive. Final RHL is determined by the interaction between a plant’s intrinsic genetic potential and its perception of and response to environmental cues. As these two components of RHL are themselves determined by cascades and networks of interacting genes, it can be predicted that the control of RHL will be complex. A substantial body of work (see Datta *et. al.*, 2011 for review) in the model eudicot Arabidopsis partially elucidates this interplay of mechanisms. However, genes have been identified that act as master regulators of final organ size for the primary root (Mouchel *et al.*, 2004; Rodrigues *et al.*, 2009) and of root hair length (*At*RSL4) (Yi *et al.*, 2010). In addition, a major quantitative trait locus (QTL) for P tolerance in rice (*Pup1*) has been shown to encode a protein kinase (Pstol1) which acts as an enhancer of early root growth (Gamuyao *et al.*, 2012), so despite the number of genetic interactions involved in determining root hair parameters it may prove that, in wheat, a few loci have a major effect.

Few QTL mapping studies for root hair traits have been undertaken. A study in the common bean (*Phaseolus vulgaris* L.) examined root hair and acid exudation traits in relation to P uptake in a recombinant inbred line (RIL) population derived from two cultivars with contrasting P efficiencies (Yan *et al.*, 2004). Longer root hairs were observed in the more efficient cultivar, with high P suppressing final hair length in both parents. Multiple QTLs were identified for both hair length and acid exudation. Examination of root hair length in a maize RIL population under P limiting conditions also found significant quantitative variation for hair length and a number of QTLs associated with root hair traits, although, interestingly, only one QTL was identified for root hair length under low P (Zhu *et al.*, 2005). A study in soyabean (*Glycine max* and *Glycine soja*) examined a number of root hair parameters, including root hair length and density in a RIL population grown on soil with moderately low P availability. The authors found that both hair length and density positively correlate with P concentration in the plant (Wang *et al.*, 2004).

Other studies have taken a broader approach, examining overall root architectural traits, but not RHL, in response to P deficiency (Beebe *et al.*, 2006; Ochoa *et al.*, 2006; Su *et al.*, 2006).

A study on root traits in wheat described a germination paper screen of root architecture, but not RHL, in seedlings of a wheat doubled haploid (DH) population derived from Savannah × Rialto (Atkinson *et al.*, 2015). The same population was grown in the field in two consecutive years. Root and field QTLs were correlated to one another, identifying QTLs for grain yield and N uptake that co-localized with QTLs for root architecture at the seedling stage (Atkinson *et al.*, 2015). In another study in wheat, a combination of approaches was used to explore variation in root traits in seedlings and yield traits in mature plants, using a DH population derived from Avalon × Cadenza (Bai *et al.*, 2013). Seedlings were grown in paper rolls in nutrient solution, and a number of root traits, but again, not RHL, were measured. In parallel, the same population was grown in the field over three years, and height and thousand grain weight (TGW) data were measured. The data sets were used for QTL analysis and the relationships between the seedling and field data explored by correlation analysis. A number of height and seed traits were coincident, with the root and seed QTLs identified on chromosomes 5A and 6A co-locating (Bai *et al.*, 2013).

In this study we introduce a method for growing wheat seedlings, imaging and measuring RHL, and then present data from experiments where this method was used to screen wheat lines for variation in hair length at the seedling stage. We tested the method in parents of eight DH mapping populations to estimate the extent of phenotypic variation and heritability of RHL in some modern wheat populations. Two pairs of mapping population parents, Spark *vs* Rialto and Charger *vs* Badger, showed clear differences in root hair length. The trait of RHL was then examined in the DH populations derived from these two crosses, in a series of consecutive experiments. RHL was measured on a total of 3925 plants. Available yield component data from 3 years of sequential field trials performed across North Europe in these two populations was used to explore the performance of the same lines under field conditions and to cross-reference the data and QTLs.

## Materials and methods

### Plant material

All preliminary root hair growth trials to develop a reproducible technique for accurate measurements of RHL were made using the UK winter wheat Riband. After the growth and screening method had been developed (see below), 16 wheat varieties, all parents of DH mapping populations, were assessed for RHL. Of the 16 lines, ten were European elite winter wheat lines, two were European elite spring lines (Highbury, Paragon), two were CIMMYT spring wheats (Weebill, Bacanora) and the final pair were Chinese Spring (a well-used experimental spring wheat line) and SQ1 (a spring wheat breeding line). These lines were obtained from the John Innes Centre (Norwich, UK) wheat collection, and were selected on the basis that the populations derived from these crosses have been used to dissect the genetic basis of a wide range of traits including yield and biotic and abiotic stress resistance. Two DH mapping populations were selected for further analysis: Spark × Rialto (119 genotypes) and Charger × Badger (95 genotypes) (Snape *et al*, 2007). Seed of all lines used were derived from field grown seed multiplication plots, 1 m^2^, sown with a Hege 90 drill, and grown to a high agronomic standard using full fungicide and herbicide treatments to ensure high quality seed.

### Grain sterilization and germination

A gas-based surface-sterilization method and an ethanol method for wheat grain coat wetting were both found to impair subsequent germination rates so we developed a water coat wetting method. Wheat grains were surface wetted and sterilized as follows: the grain were placed in a 20ml vial, and covered with 10ml of autoclaved water. The contents were vortexed for 30s to remove air bubbles from the grain surface, and then the vials were placed on a table agitator for an hour at room temperature. One millilitre of 20% sodium hypochlorite was added to each tube, the contents mixed using a vortex mixer and left for 10min. The vials were drained and the grain rinsed thrice. Grains were transferred to sterile Petri dishes lined with moist filter paper, placed so that they did not contact each other. The dishes were sealed with Micropore™ (3M) tape and transferred to an incubator set at 28 ºC, and the contents moistened every 24h. Germination normally occurred between 48 and 72h from sterilization.

### Growth method

Square plates of 100ml were used for subsequent growth of the germinated grain; 80ml of growth medium (Murashige and Skoog basal salts 2.15g l^–1^, Phytagel 2g l^–1^; Sigma-Aldrich, Poole, UK) was dispensed per plate. Cellophane discs (80mm diameter; AA packaging, Preston, UK) were trimmed to give a straight edge on one side and autoclaved in water. One disc was placed on top of the firmed medium in each plate, with the straight edge parallel to and about 25mm from one edge. Five germinated grain were place on each plate equidistant from each other on the straight edge of the cellophane, with the shoot pointing upwards, and the grain body lightly embedded into the medium; the lids were replaced and the plates were sealed with Micropore. This alignment meant that the root grew down over the cellophane and not into the medium. The plates were tilted, seedling side up, against a frame set at an angle of 35º off the vertical, and taped together in batches of five so that the plates maintained this angle. The plates were placed in the growth chamber in a random order and rearranged arbitrarily every 24h.

### Imaging and image analysis

Images were captured at ×8 magnification using a stereomicroscope coupled to a digital camera (Nikon CoolPix 5700). Images were taken through the underside of the plate for sharp contrast, reduction of scattered light from medium surface, and a constant focal distance from microscope lens to the roots growing on the medium surface. Images were captured using dark field microscopy with a strong directional light applied to one side of the plate. The images were saved as JPEG files. The images were processed using the software package ImageJ (National Institutes of Health, https://imagej.nih.gov/ij/). Each batch of images included an image of a piece of graph paper for calibration purposes. The program ImageJ was recalibrated to this internal control as each batch of images was processed

### Root hair measurements

**Fig. 1.**
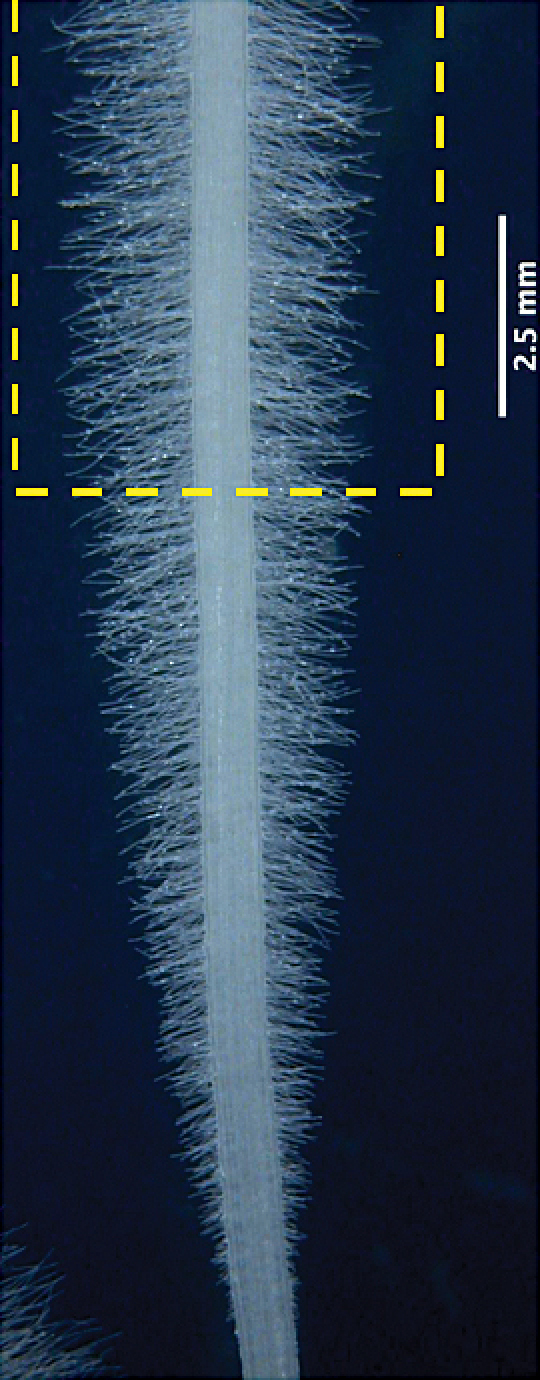
Wheat seedling root 3 days post-germination. Yellow box indicates mature root hair zone.

Only root hairs within the mature zone, as indicated by the box in Fig. 1, were analysed. Root hairs in this zone had reached their maximum length. Measurements were made by drawing and taking the length of a line from the beginning to the end of each of the five apparently longest hairs in each image, using the measuring tool in ImageJ. Data were recorded in an Excel spreadsheet.

### Experimental design for DH population measurements

Sixteen wheat DH mapping population parents were screened for between-parent variation in RHL at the seedling stage. Twelve grains from each line were surface sterilized and germinated. Ten seedlings per line were then transferred to nutrient medium plates, with five seedlings on each of two plates per line. Two of the DH mapping populations had parents that differed significantly for root hair length (see Results). The bi-parental mapping populations derived from these parents, Spark × Rialto (115 DH lines) and Charger × Badger (95 DH lines), were used for further study. All DH lines from both populations were used, but in each experiment several of the lines failed to germinate or succumbed to fungal infection. For each DH line, six grain were sterilized and germinated. Five of the seedlings were plated on a single plate, to give one plate per line per experiment. The Spark × Rialto population was examined in five consecutive experiments, whilst the Charger × Badger population was studied twice. The root hairs were measured and recorded as described above.

### Statistical analysis

Statistical analysis was performed in R software (v. 3.1.1) (R core team 2013). A linear model was fitted to the RHL data for each population, with the experiments as cofactors, using function ‘lmer’ of the lme4 package (v. 1.1-7). A similar linear model was fitted to the Charger × Badger RHL data. Significant genotype and experiment effects were detected and also significant genotype × experiment interactions. The model explained about 80% of the experimental variation. For both populations the best linear unbiased predictors (BLUPs) were calculated from the RHL data and used for QTL detection and correlation with the field data.

Estimates of narrow-sense heritability (*h* ^2^_ns_) of RHL in each DH mapping population were determined from variance components using expected mean squares as follows:

$$h^{\text{2}}{}_{\text{ns}}\;=\;\sigma^{\text{2}}{}_{\text{g}}/(\sigma^{\text{2}}{}_{\text{g}}+\sigma^{\text{2}}{}_{\text{e}})$$

where: σ^2^_g_ is genotypic variance (σ^2^_g_ = (MS_lines_ – MS_error_)/*k*, where *k* is number of replications and MS is mean square) and σ^2^_e_ is environmental variance (σ^2^_e_ = MS_error_).

#### Field trial data

Field data for the populations Spark × Rialto and Charger × Badger were generously provided by the authors of Snape *et al.* (2007) and (Simmonds *et al.*, 2014). In summary, the two populations were grown in randomized and repeated sites in Scotland, England, France and Germany as well as at the John Innes Centre trial site at Church Farm, Bawburgh in the years 2001, 2002 and 2003. A number of physiological traits were recorded for each line

#### QTL analysis

Previously derived genetic maps for the populations Charger × Badger and Spark × Rialto (Snape *et al.*, 2007) were used for QTL analysis. For the QTL analysis the R package ‘qtl’ (v. 1.29) was used. A single QTL model was fitted as an initial QTL scan along the chromosomes, employing the extended Haley–Knott method on imputed genotypes. Significance thresholds for QTL detection were calculated from the data distribution. Final QTL LOD scores and effects were received from a multiple QTL model where more than one QTL was detected in the initial scan.

## Results

### RHL variation in the parent lines

**Fig. 2.**
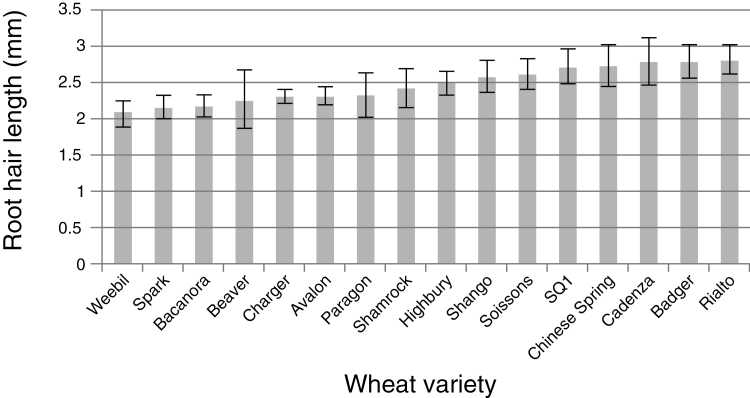
Bar graph of mean root hair length for the 16 mapping population parental lines. Error bars indicate standard deviation.

In order to identify DH populations segregating for RHL, the trait was examined in 16 DH mapping population parental lines. The mean RHLs of the 16 varieties examined are shown in Fig. 2. Of these, three had average hair lengths of between 2.0 and 2.30mm (Weebill, Bacanora and Spark) whilst others had much longer hairs, with lengths in the range 2.70–3.10mm (Rialto, Badger, and Chinese Spring), with the remainder of the varieties examined having RHL intermediate between these values. For several of the parental pairs of available mapping populations, RHL did not significantly differ between the two parents. However, two populations were identified that showed significant differences for the trait. These were Spark × Rialto, *t*=6.37, *P*<0.001, and Charger × Badger, *t*=6.80, *P*<0.001.

### RHL is normally distributed and shows transgressive segregation in the DH populations Spark × Rialto and Charger × Badger

In the segregating DH populations Spark × Rialto and Charger × Badger, the data for RHL showed a normal distribution in each experiment (data not shown). Additionally, transgressive segregation was observed in both directions beyond the parental values (data not shown) indicating the polygenic nature of the trait. The descriptive statistics for the performance of each DH population are given in Table 1.

**Table 1.** Descriptive statistics for the Spark × Rialto and Charger × Badger mapping populations

	**Average root hair length (mm**)						
	**Parental lines**		**DH lines**				
	**Spark**	**Rialto**	**Min**	**Max**	**Mean**	**Median**	**SD**
Exp. 1	1.93	2.59	1.20	2.89	2.15	2.16	0.22
Exp. 2	2.11	2.66	1.65	2.94	2.11	2.09	0.18
Exp.3	2.06	2.73	1.56	3.00	2.31	2.33	0.25
Exp. 4	1.85	2.68	1.61	2.99	2.37	2.37	0.21
Exp. 5	2.17	2.79	1.78	3.17	2.49	2.49	0.25
	**Charger**	**Badger**	**Min**	**Max**	**Mean**	**Median**	**SD**
Exp. 1	2.30	2.76	1.60	3.20	2.35	2.34	0.29
Exp. 2	2.27	2.82	1.62	3.12	2.47	2.48	0.29

### Significant experiment × genotype interactions were observed in both populations

For the Spark × Rialto population, no significant genotype effect was detected; however, there was a significant experiment effect and a significant experiment × genotype interaction. Due to the high level of variability within the datasets, only about 30% of the experimental variation could be explained by the model. However RHL in Charger × Badger showed significant differences between genotypes and the unexplained variation (residual sum squares) was approximately 3 times lower than the genotypic variation. This is reflected in the narrow-sense heritabilities. For RHL in Spark × Rialto, *h* ^2^_ns_ was 0.59, whilst for Charger × Badger it was higher, at 0.83.

The detected experiment × genotype interaction indicates that there is considerable variation in the way each line responds in each experiment, consistent with the extreme environmental sensitivity of root hair growth. The rank order of the DH lines by hair length (not shown) is not the same from one experiment to the next, with some lines showing more variation than others. However, analysis of the data for the 50th longest measurements in each experiment showed that a number of lines were consistent in their response to their environment.

Calculation of the coefficient of variation (CV; SD/mean×100%) gave values of 8.9% for Spark × Rialto and 8.6% for Charger × Badger. These similar values are derived from the corrected RHL for both populations and indicate that the power of QTL detection would be similar in Charger × Badger and Spark × Rialto. However, as the Charger × Badger population contained fewer genotypes (95) than the Spark × Rialto population (119), we were more likely to detect QTLs in the latter.

### Identification of QTLs associated with variation in RHL

In the population Charger × Badger one significant QTL for RHL was detected on chromosome 2B, closest marker BS00009247 (3). The 2B QTL had a LOD score of 3.8, explaining 17.6% of the observed variance, with an additive increasing effect of 0.72mm coming from Charger.

**Fig. 3.**
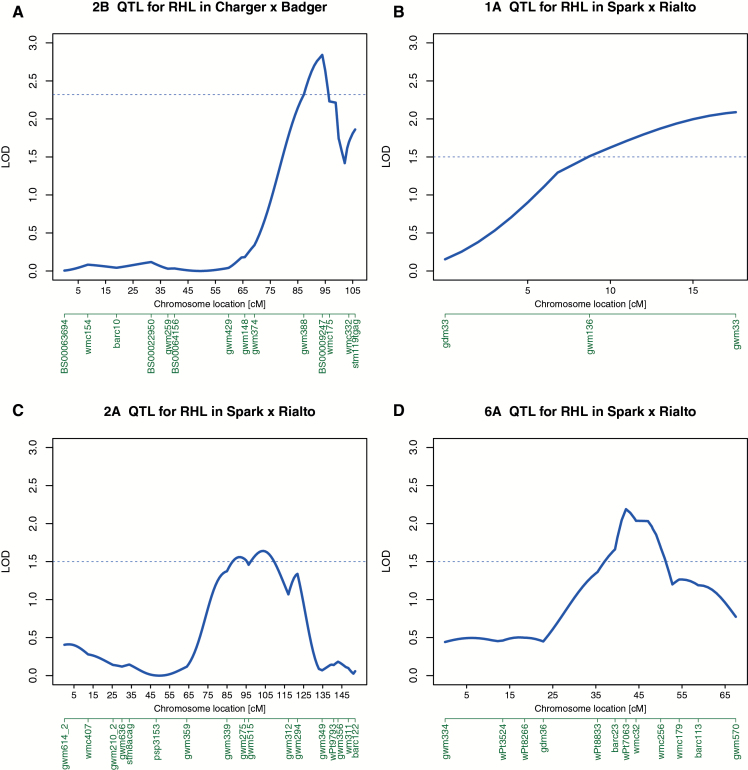
Plots of root hair length (RHL) QTL LOD scores along selected chromosomes. Marker names are given below the chromosome location axis. The dotted horizontal line shows the signiﬁcance threshold. (A) 2B QTL in Charger × Badger; (B) 1A QTL in Spark × Rialto; (C) 2A QTL in Spark × Rialto; (D) 6A QTL in Spark × Rialto.

In Spark × Rialto three significant QTLs were detected, on chromosomes 1A, 2A and 6A (see Fig. 3). The 1A QTL, at Xgwm33, had a LOD score of 2.7, explaining 8.2% of the variance, and had an additive increasing effect of 0.28mm, coming from Spark. The 2A QTL, at Xgwm515, had a LOD score of 1.9, explaining just 5.7% of the variation with the increasing allele coming from Rialto. Finally, the 6A QTL, at wPt7063, had a LOD score of 2.4, explaining 7.2% of the variation, with the increasing allele coming from Rialto.

### Correlation between RHL and yield traits

In order to investigate whether there was a relationship between root hair length and increases in yield components scored in the field, correlation analyses between the data sets for thousand grain weight (TGW), harvest index (HI) and yield and the data sets for RHL were performed as a Pearson product-moment correlation, using the ‘cor’ function from the ‘R base’ package. Significant (≥95% confidence level) correlations were observed between RHL and either TGW or yield in 7 out of 16 pair-wise comparisons for Spark × Rialto (Table 2). In the Charger × Badger population (Table 2), correlation analysis between RHL and field traits showed that in 6 out of 15 pairwise comparisons, there was significant correlation between RHL and either yield or TGW.

**Table 2.** Correlations between root hair length means and mean data from field trials for the Spark × Rialto and Charger × Badger populations

**Site**	**Year**	**Yield component**	**Charger × Badger**		**Spark × Rialto**	
			**Spearman’s rho**	***P*-value**	**Spearman’s** **rho**	***P*-value**
Germany	2002	TGW	0.20	0.07	0.07	0.46
		YLD	0.27	0.01368*	0.05	0.59
	2003	TGW	0.21	0.0495*	0.18	0.05
		YLD	−0.02	0.87	0.04	0.65
Scotland	2002	TGW	0.21	0.06	0.22	0.016*
		YLD	—	—	0.24	0.00739**
	2003	YLD	0.21	0.05	0.25	0.0055**
France	2002	TGW	0.24	0.0266*	—	—
		YLD	0.19	0.07	0.18	0.0483*
	2003	YLD	0.05	0.62	—	—
		TGW	0.30	0.00531**	0.21	0.021*
Church Farm, UK	2001	TGW	—	—	0.25	0.007**
		YLD	−0.01	0.92	0.18	0.05
	2002	TGW	0.24	0.0248*	0.12	0.18
		YLD	0.11	0.30	0.10	0.30
	2003	TGW	0.21	0.0452*	0.26	0.005**
		YLD	0.10	0.37	0.13	0.17

*Significant at the 5% confidence level; **Significant at the 1% confidence level. TGW, thousand grain weight; YLD, grain yield.

## Discussion

The presented method developed for RHL measurement allows for rapid screening of large numbers of lines, and uses easily available lab equipment and a freely available image processing package. The aim of the method development was to establish a highly reproducible and efficient growth method, reducing environmental variation as far as possible. The well reported plasticity of root hair growth was confirmed by our own trials in soils (data not shown). Seedlings grown in soil between glass plates showed 2- to 3-fold changes in RHL along the root, depending on the microenvironment surrounding the root. As the aim of this investigation was to compare hair lengths between genotypes, it was desirable to remove as much experimental variation as possible. For this reason we chose an artificial growth system.

A number of root growth systems are reported in the literature, including using germination paper, growing in pots (Bates and Lynch, 2000*a*; Haling *et al.*, 2013, 2014; Delhaize *et al.*, 2015), in baskets in pots (Hamada *et al.*, 2012), deep root trainers (George *et al.*, 2014), on Turface clay (Goron *et al.*, 2015), in germination pouches (Atkinson *et al.*, 2015; Miguel *et al.*, 2015), in hydroponic systems (Petrarulo *et al.*, 2015), on agarose (Han *et al.*, 2015), and in plant growth ‘microcosms’ (Keyes *et al.*, 2013). In our system, the root hairs are significantly longer than those reported for soil-grown wheat and barley (e.g. Haling *et al.*, 2013, 2014; Delhaize *et al.*, 2015), but of the same order of length as reported for medium-grown wheat (Han *et al.*, 2015). Medium-grown roots are exposed to air on one side of the root, promoting maximum elongation, which likely explains the difference in hair length observed between soil-based and medium-based experiments. Gahoonia and Nielson (1997) reported that in barley, the observed rank order of RHL by cultivar under laboratory conditions remained constant when the same cultivars were grown in the field. However, soil strength can also have an effect on hair elongation: in a study on barley, high-strength soil caused a 20% reduction in root hair length, although even under supressing conditions, root hairs were still found to have a benefit in terms of P uptake (Haling *et al.*, 2013). Future studies should look at the effect of soil strength on the expression of the longer haired phenotype in both Charger × Badger and Spark × Rialto.

It was necessary to develop a surface sterilization protocol that did not include an ethanol rinse as this seemed to inhibit germination, as the initial trials with cultivar Riband demonstrated (data not shown). Elsewhere, it has been shown that the quantity of endogenous ethanol released from waterlogged grain correlated with reduced germination rates in a cultivar-specific manner (Ueno and Takahashi, 1997), so it remains possible that the cultivar used in our initial trials was particularly sensitive to ethanol.

To assess root hair length, the roots had to be prevented from growing into the medium. To achieve this, the medium surface was covered in cellophane discs. Cellophane may influence root hair growth. Experiments with root hairless mutants have shown that the double mutant *rhd6*/*rsl1*, which has no root hairs, can be complemented with *RSL1*, but root hair growth was only restored, if the transformants were grown on medium containing either ethylene or auxin, or on cellophane (Menand *et al.*, 2007). The influence of cellophane could not be tested in our experimental set-up, as the omission of the cellophane would have resulted in much larger errors in the root hair length measurements. We assumed a uniform or minor influence of cellophane on the growth level as it was used in all of our experiments consistently, but we could not easily test this assumption.

One factor that was not considered when testing the method was the effect of grain and embryo size on seedling root growth. It has been reported that variation in the size of the seed positively correlates with initial root development (Bremner *et al.*, 1963; Evans *et al.*, 1977). This in turn may have had an effect on the development of root hairs. However, the grain used in our trials and experiments were taken from bulked samples, from different years, so it may be assumed that the grain and embryo sizes are representative of the normal range for each genotype.

The correlation analysis showed that there are positive correlations between variation in yield traits and root hair length in both mapping populations in different sites and years. This supports the hypothesis that root hairs are beneficial in the field. Unfortunately environmental data were not collected for the field trials, so we are unable to conclude when RHL is correlated with increased yield, as indicated by correlation with yield components, in some sites and years but not others.

A genetic analysis of rhizosheath size, which is largely determined by root hair length, identified six major loci for the trait, including QTLs on chromosomes 2BL and 6AL (Delhaize *et al.*, 2015). Another study in wheat grown on low P soil identified nine QTLs for shoot biomass under limiting conditions, including QTLs on 1A, 2B and 6A. The Spark × Rialto datasets used here for correlation analysis between field and RHL traits have been described in detail in Snape *et al.* (2007). After initially observing a significant, novel QTL for yield and TGW on 6A (Snape *et al.* 2007), the authors increased the marker resolution across the region and reanalysed the phenotypic data, using a multi-trait multi-environment (MTME) to more precisely position the QTL. They identified the yield QTL, *Qyld-jic.6A*, as significant in 9 out of 12 environments, with the peak at *wPt-7063* (Simmonds *et al.*, 2014). As with the Spark × Rialto QTL on 6A for RHL, it was Rialto that provided the beneficial allele. Snape *et al.* (2007) also reported that the yield QTL on 6AL associated with *wPt-7063* also segregate in other populations: in the cross Spark × Rialto, it is the Rialto allele that increases performance, whilst in the cross Savannah × Rialto it is the Savannah allele that has a yield increasing effect.

Simmonds *et al.* (2014) describe an MTME analysis of the Spark × Rialto data showing yield, green canopy duration and TGW effects, stable across a number of North European environments, all co-localized to an 8 cM region between *wPt-7063* and *Xgwm256.* Near isogenic lines (NILs) segregating for this QTL cluster were developed and trialled over 5 years at sites in England and Germany. The Rialto 6A NIL significantly increased TGW (2011, 2012, and 2013), and significantly increased yield (2010, 2012 and 2013). However, there was a strong environment interaction: in 2012 no effect on TGW was observed in the Rialto NILs and there was a negative effect on yield. The authors observed that this year had an unusually warm and dry start to the growing season followed by a particularly wet summer. A recent study in wheat (Bai *et al.* 2013) measured both root traits in seedlings and yield components in mature plants, in the DH mapping population Avalon × Cadenza. QTLs for a number of traits were coincident. Total root length and seminal lateral root area were reported as associated with the marker *wPt-7063* on chromosome arm 6A, QTL for TGW. Together, these studies show that the region surrounding the marker *wPt-7063* on 6A is involved in the control of a number of root traits including RHL as well as yield components, and is of particular interest for further study. There was no co-localization between the QTL for RHL on 2AL in Spark × Rialto and other field traits, so it is possible that it is not expressed under field conditions. The QTL described on 2AL in Spark × Rialto and 2BL in Charger × Badger may be homoeoallelic, differentially segregating in the two genetic backgrounds. An *in silico* analysis to determine the relationship between the regions surrounding the two loci proved inconclusive, as did COS and PLUG marker approaches to fine map the region, so this hypothesis remains to be resolved.

Determining which genes underlie the QTLs remains a challenge for the future. However, in *Arabidopsis*, a family of bHLH transcription factors has been shown to be involved in root hair development. This family includes *AtRHD6* and a highly similar gene, *AtRSL1*, whose orthologues are also involved in rhizoid development in the moss *Physcomitrella patens* (Menand *et al.* 2007). Conservation of function from an ancient mutual progenitor supports the probability that this family of genes will be found to function in determining RHL in wheat. Overexpression analysis of another member of this family, *AtRSL4*, resulted in root hairs that were significantly longer as a result of a longer period of growth (Yi *et al.*, 2010), and it has been proposed to act as a master regulator of root hair length. Overexpression of a wheat orthologue of this gene, *TaRSL4*, also resulted in longer root hairs, and subsequent increase in biomass under nutrient poor conditions (Han *et al.*, 2015).

Understanding the control of RHL could have important implications for developing wheat with improved drought avoidance and tolerance, as well as for enhanced nutrient uptake. To date there are few QTL mapping studies on root hair traits, and none in wheat. This first study demonstrates that there is a relationship between longer root hairs and yield components, and thus the potential usefulness of examining this component of water and nutrient uptake. Further work needs to be done to dissect the QTLs on wheat Group 2 and 6 in order to establish which loci would be of particular interest for developing markers for breeding programmes. Fine mapping the region using recombinant inbred lines would be a useful strategy to help determine the control of the different effects and the extent of pleiotropy between root and grain traits.

## Abbreviations:

DH: doubled haploid
NIL: near isogenic line
QTL: quantitative trait locus;
RHL: root hair length
TGW: thousand grain weight.
